# Research progress and applications of nanobody in human infectious diseases

**DOI:** 10.3389/fphar.2022.963978

**Published:** 2022-08-12

**Authors:** Yaxian Mei, Yuanzhi Chen, Jwala P. Sivaccumar, Zhiqiang An, Ningshao Xia, Wenxin Luo

**Affiliations:** ^1^ State Key Laboratory of Molecular Vaccinology and Molecular Diagnostics, School of Public Health, School of Life Science, National Institute of Diagnostics and Vaccine Development in Infectious Diseases, Xiamen University, Xiamen, China; ^2^ Texas Therapeutics Institute, Brown Foundation Institute of Molecular Medicine, University of Texas Health Science Center, Houston, TX, United States

**Keywords:** nanobody, single-domain antibody, human infectious diseases, antibody engineering, therapeutic

## Abstract

Infectious diseases, caused by pathogenic microorganisms, are capable of affecting crises. In addition to persistent infectious diseases such as malaria and dengue fever, the vicious outbreaks of infectious diseases such as Neocon, Ebola and SARS-CoV-2 in recent years have prompted the search for more efficient and convenient means for better diagnosis and treatment. Antibodies have attracted a lot of attention due to their good structural characteristics and applications. Nanobodies are the smallest functional single-domain antibodies known to be able to bind stably to antigens, with the advantages of high stability, high hydrophilicity, and easy expression and modification. They can directly target antigen epitopes or be constructed as multivalent nanobodies or nanobody fusion proteins to exert therapeutic effects. This paper focuses on the construction methods and potential functions of nanobodies, outlines the progress of their research, and highlights their various applications in human infectious diseases.

## Introduction

Microorganisms play an important role in our ecosystem, yet some microbes like viruses, bacteria and fungi or its toxic products tend to be pathogenic by both direct and indirect means which impose global healthcare burden. According to the survey published by the World Health Organization, millions of deaths were accounted due to the communicable diseases just in the year. In addition to persistent communicable diseases like malaria, dengue, human immunodeficiency virus (HIV), Human papilloma virus (HPV) and Hepatitis B virus (HBV), an emergence of outbreaks like Spanish Flu, Ebola, Zika, SARS- CoV-2 further challenges the advancement of science and increases the morbidity and mortality rate. To note, the novel coronavirus pathogen (SARS-CoV-2), which emerged in 2019 has already accounted for more than 6 million deaths globally as of June 1st, 2022 (WHO coronavirus (COVID-19) dashboard. https://covid19.who.int/). This demands the requirement of new diagnosis and treatment strategies in addition to the already available anti-microbial drugs.

The advancement of technology has allowed for the development of various tools in scientific research that have opened a therapeutic window. One such advancement includes the rise of antibody-based immunotherapy. Antibodies (Abs) are regarded as an attractive biomolecule for the diagnosis and therapy of infectious diseases due to its target specificity, structural flexibility and diversity. Among the different formats of antibody-based molecules, nanobodies (Nbs), emerged as suitable candidate as a diagnostic agent as well as therapeutics in treating infectious diseases (Table 1). Nanobodies, as natural small molecule antibodies, have attracted attention and play an increasingly important role in treating infectious diseases. Several nanobodies for infectious diseases are currently being tested in clinical research, such as MucoRice-ARP1 for rotavirus (Sarker et al., 2013; Tokuhara et al., 2013) and ALX-0171 for respiratory syncytial virus (RSV) (Cunningham et al., 2021). During the COVID-19 epidemic, a number of nanobodies targeting SARS-COV-2 with high affinity have been screened and constructed into different forms, such as bivalent form (Ma et al., 2021), bispecific form (Xiang et al., 2020), trivalent form (Dong et al., 2021) and so on, demonstrating good therapeutic effects *in ex* or *vivo* experiments (Xiang et al., 2020; Huo J. D et al., 2021; Ma et al., 2021).

**TABLE 1:** Representative nanobodies for infectious diseases.

Nanobody	Disease	Target	Structure features	References
MR3	SARS-CoV-2	RBD	Bivalent Nb; bispecific Nb; VHH-Fc	Li et al. (2021)
D7,D3	*Ehrlichia* infection	T4SS effector	CPP-Nb conjugation	Zhang et al. (2021a)
CeVICA	SARS-CoV-2	RBD	Nb-His tag	Chen et al. (2021)
Fu2	SARS-CoV-2	RBD	Bispecific; bivalent; trivalent; VHH-Fc	Hanke et al. (2022)
125s	chronic hepatitis B infection	hepatitis B surface antigen	VHH-Fc	Wang et al. (2022)
/	Fungal Keratitis	Dectin 1	Nb-His tag	Liu et al. (2022)
VUN100	Latent human cytomegalovirus (HCMV) infection	signaling of the viral receptor US28	Nb–photosensitizer conjugates; bivalent Nb; Nb-His tag	De Groof et al. (2019); De Groof et al. (2021)
Nb113 and IB10	Shiga toxin-producing *Escherichia coli* (STEC)	the B subunit of Shiga toxin 2 (Stx2B) and the C terminus of Intimin (IntC280)	Bispecific Nb	Bernedo-Navarro et al. (2018); Ruano-Gallego et al. (2019)
Nb13	Mal de Río Cuarto virus (MRCV)	the major viral viroplasm component, P9-1	Nb-alkaline phosphatase; Nb-eGFP fusion	Llauger et al. (2021)
NB7-14	Influenza H7N9 virus	HA	Bivalent Nb	Gaiotto et al. (2021)
C5, H3, C1, F2	SARS-CoV-2	RBD	Homotrimers Nb; VHH-Fc	Huo J et al. (2021)
aEv6	Ebola Virus	EBOV GP	Nb-Fc	Esmagambetov et al. (2021)
aRBD-2, aRBD-3, aRBD-5, aRBD-7, aRBD-41, aRBD-42, and aRBD-54	SARS-CoV-2	RBD	Bispecific Nb; Nb-His tag; Nb-Fc	Ma et al. (2021)
Nb1	bovine viral diarrhea virus	The nonstructural protein 5	Nb-eGFP fusion	Duan et al. (2020)
NbCXCR4	human immunodeficiency virus	CXCR4	Fuse Nb to an anti-fluorescein (FITC) scFv responsible for carrying the FITC-labeled siRNA	Jähnichen et al. (2010); Cunha-Santos et al. (2020)
NbMS10	Middle East respiratory syndrome (MERS) coronavirus (MERS-CoV)	RBD	bivalent Nb	He et al. (2019).Zhao et al. (2018)
			trimer Nb	
			Nb-His tag	
			Nb-Fc	
SNB02	Severe fever with thrombocytopenia syndrome virus* (SFTSV)	The extracellular domain of SFTSV Gn (sGn)	Nb-Fc	Wu et al. (2020)

In this study, we demonstrated the construction methods and potential functions of nanobodies and summarized their recent progress in infectious diseases.

### Characteristics of nanobody

Nanobodies were first discovered in camelids by Hamers-Casterman and his team in 1993. They found that there are heavy-chain-only antibodies (HCAbs), comprising a CH2 constant domain, a CH3 constant domain, a hinge region, and a variable heavy chain domain (VHH), in camelids (Hamerscasterman et al., 1993)**.** The VHH is also called as nanobody. Compared to the typical antibody with the weight of 150 kDa, antibody fragments like Fabs (50 kDa) and scFv (25 kDa), nanobodies are smaller in size (15 kDa) (Salvador et al., 2019). They are the naturally smallest single domain antibodies that have been identified. The lack of a light chain simplifies posttranslational modifications of nanobodies and contributes to diverse expression systems (Salvador et al., 2019). Nanobodies are formed by four framework regions (FR1, FR2, FR3, FR4) and three complementarity determining regions (CDR1, CDR2, CDR3). CDR3, which contributes to antigen binding in nanobodies, is on average longer and has increased amino acid residue variability than conventional Abs. Therefore, nanobodies bind with high affinity and can recognize more epitopes, particularly some “hidden”, cryptic epitopes (De Genst et al., 2006; Steeland et al., 2016; Zavrtanik et al., 2018). Moreover, there are four hydrophilic amino acid residues in FR2 and a disulfide bond between CDR1 and CDR3. These unique structures provide them with high solubility and good stability under extreme pH or temperature. These structures are beneficial to drug conservation and delivery and offer the possibility of oral or nasal drug uptake routes for nanobodies (Dumoulin et al., 2002; Li et al., 2015; Omidfar and Daneshpour, 2015). The immunogenicity of antibodies may cause serious side effects *in vivo*, especially when they are used in high doses or large amounts. Humanization can effectively reduce the immunogenicity of antibodies while retaining the specificity and affinity, which contributes a lot to the applications of therapeutic antibodies (Almagro and Fransson, 2008). Compared to traditional antibodies, nanobodies exhibit high homology with human VH domains, which translates to easier human source transformation and in turn decreases clinical side effects. The conventional way of humanizing VHH is to replace the framework residues on the target VHH with corresponding residues of human VH, avoiding the substitution of hydrophilic amino acids on VHH FR2 (Phe42, Glu49, Arg50, and Gly52) (De Genst et al., 2004). Humanization of nanobodies can also be achieved through straightforward site-directed mutagenesis considering that the gene contains only approximately 380 bp (Vincke et al., 2009; Salvador et al., 2019). Vincke C and his colleagues have humanized VHH by replacing 12 amino acid residues on VHH that differ from the corresponding region of the human VH (Vincke et al., 2009). In conclusion, these advantages render nanobodies promising for infectious diseases.

### Construction of nanobody library

**FIGURE 1:**
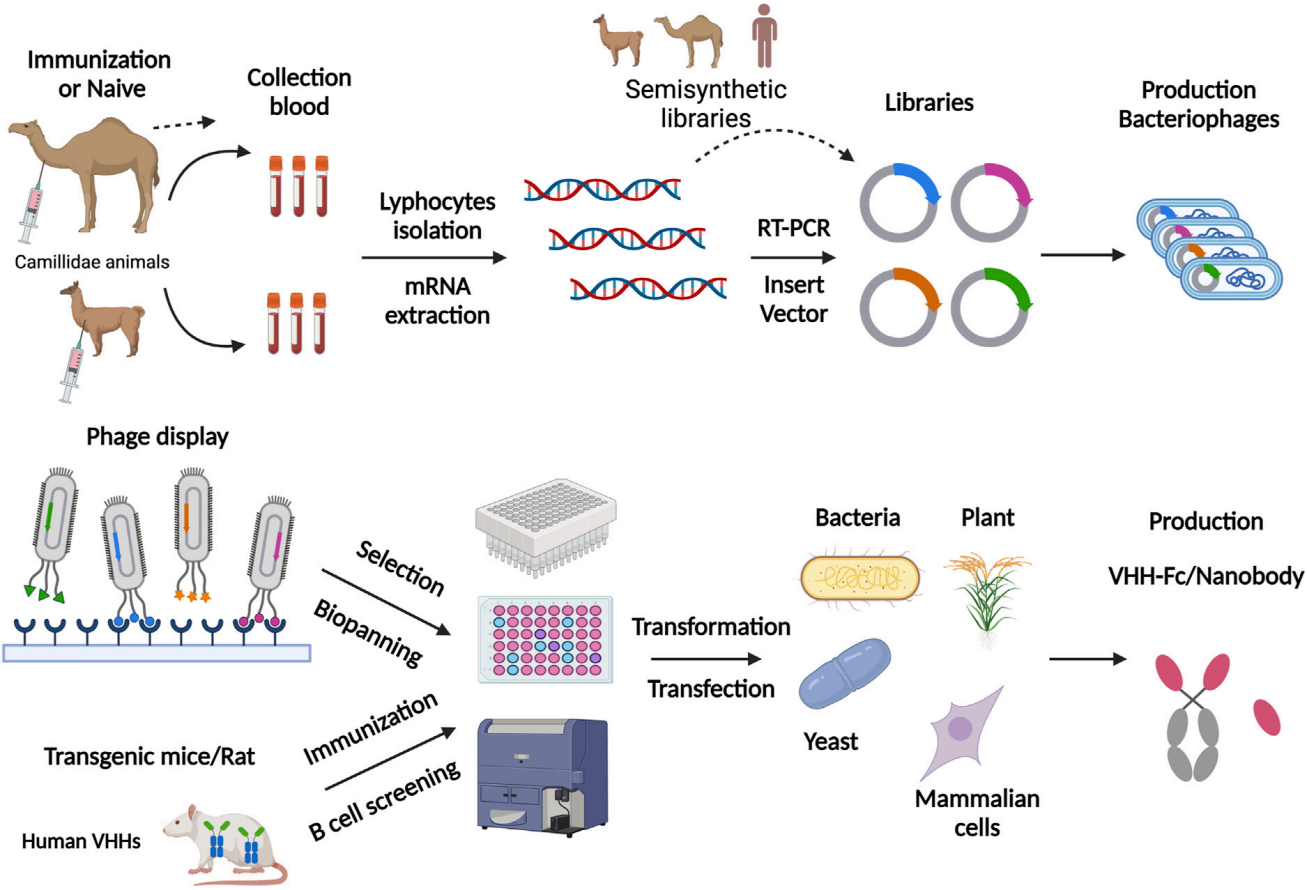
Schematic representation of nanobody production scheme through phage display library and nanobody transgenic animals. For phage display, this figure involves the steps of immunization, library preparation, cloning into the phagemid vector, and transforming into a suitable *E. coli* strain. They are subsequently panning on the target antigen, followed by the washing and elution steps. For nanobody transgenic animals, the experimental stages involved were immunization, direct B cell screening and cloning, followed by *in vitro* screening. Finally, antigen-specific nanobodies are produced in various expression systems, such as plants, bacteria, yeast and mammalian cells. Figure 1 was created with BioRender.com.

Most antigen-specific nanobodies are obtained from constructed libraries. Nanobodies can be generated from immune, naïve, or synthetic VHH libraries (Liu W S et al., 2018). Immune libraries are the most effective approach for obtaining functional nanobodies. As the Figure 1 shows, to construct immune libraries, Camillidae animals, like dromedary, alpaca, llama and Heavy Chain Only Antibodies (HCAbs) transgenic mice are actively immunized with a special immunogen (Janssens et al., 2006; Liu and Yang, 2022). These immunogens stimulate the immune system to raise HCAbs with reasonable specificity and high affinity (De Genst et al., 2004). Next, lymphocytes from the host are isolated to extract mRNA. Then, cDNA fragments are obtained from reverse transcription, and VHH sequences are obtained and amplified through nested Polymerase Chain Reactions (PCRs). The unique VHH sequences are inserted into a vector and transformed into an expression system for the next screening. Nb library with >10^7^ individual transformants is considered to be of good quality for the next screening (Muyldermans, 2021). Naïve libraries are more convenient because they depend on the natural immunological diversity in camelids and do not require active immunization. In addition, they allow for identification of nanobodies binding to any specific antigens, such as non-immunogenic antigens or toxic antigens (Yan et al., 2015). However, the size and diversity limitations of libraries preclude high efficiency screening of nanobodies. Naïve libraries should have 10^9^–10^10^ individual clones, which rely on massive amounts of blood (probably >10 L) from different animals to avoid a bias against particular antigen in one animal (Muyldermans, 2021). Synthetic libraries, which rely on targeted mutagenesis in special CDR regions, yield sufficiently diverse libraries for targeted nanobodies. screening (Muyldermans, 2013; Liu W S et al., 2018 **)**. Synthetic libraries, like naïve libraries, also allow the screening for nanobodies targeting specific antigens without immunization. However, synthetic libraries require a more complex and efficient library building design, and a larger library quality of 10^9^–10^15^ to screen for the targeting nanobodies (Muyldermans, 2021). Liu and Yang summarized a systematic approach to construct synthetic libraries, including the selection of suitable framework sequences, the design of CDRs’ randomization, and the synthesis of a sufficient number of DNAs with different sequences. The undesirable frameshift mutation can be controlled through optimizing the primer quality, the DNA polymerase fidelity, and experimental techniques, in order to improve the quality of synthetic libraries (Liu and Yang, 2022).

However, due to the diversity of libraries or other limitations, suitable nanobodies are difficult to obtain. In this situation, selected nanobodies are considered to improve affinity through molecular techniques, such as error-prone PCR, DNA family shuffling and specific amino acid mutations (Koide et al., 2007; Steeland et al., 2016; Lecocq et al., 2019 **).**

### Expression system of nanobody

For the production of nanobodies, there are two primary expression systems, prokaryotic and eukaryotic (Figure 1). The most common choice is bacteria expression systems like *E. coli.* due to its easy purification and low cost (Makvandi-Nejad et al., 2011). Moreover, yeast and fungal expression systems are suitable for a high production of nanobodies (Ezzine et al., 2012; Gorlani et al., 2012; Steeland et al., 2016). Mammalian cells expressions, such as HEK293T cells, CHO cells, and Expi293F, are good choices for producing of VHH-Fc fusions, Nbs-HRP fusions and nanobodies in bivalent or multivalent forms (Steeland et al., 2016; De Marco, 2020). Ario elaborated on the examples of nanobodies expressed in Mammalian cells expressions (De Marco, 2020). The Nb-fusion protein can also be expressed in bacteria when the Nb is fused just for providing a tag to facilite purification (Djender et al., 2014; Liu Z. H et al., 2018).

Different expression systems facilitate nanobodies screening with different approaches. Phage display technique based on *E. coli* expression systems is the most common screening method. F*v* genes are constructed into the phage genome and displayed on the phage surface together with phage coat protein (Liu and Yang, 2022). Phages are secreted by *E. coli.*, and they can bind to targeted antigens on solid surfaces based on the affinity of nanobodies. In this procedure, phages are amplified and screened consecutively to enrich and identify suitable antigen-specific phages, which may take lots of time and effort. *In vitro* assays, such as phage ELISA, are used to identify phages displaying nanobodies with good affinity (Hoogenboom, 2005; Liu W S et al., 2018). Besides, other display platforms based on different expression systems are used for nanobody screening. Conor and his colleagues generated nanobodies targeting human G-protein-coupled receptors (GPCRs) through a constructed yeast surface display platform (Mcmahon et al., 2018). A cell-free Nb engineering platform based on ribosome display technology was successfully applied to screen nanobodies against Receptor-Binding Domain (RBD) of SARS-CoV-2 (Chen et al., 2021).

Diverse expression systems also allow for other avenues of nanobodies applications. Some pathogens are transmitted through the gastrointestinal or genital tract and cause serious diseases. For people at high risk of these diseases, passive immunity is considered an effective protective measure. Nanobodies has been shown to maintain functional activity under extreme conditions and are resistant to pepsin. They perform well in the digestive and genital tracts. By taking foods such as *Arabidopsis thaliana* seeds and rice that express nanobodies, patients with the related diseases can be effectively protected.

Researchers constructed an expression system of transgenic rice to express anti-rotavirus specific nanobodies (MucoRice-ARP1). MucoRice-ARP1 is already in clinical trials currently, which displays good water solubility, stability and high expression levels (rice expression system). It promises to be an attractive, ready-to-use, oral, anti-rotavirus product for mass production applications. MucoRice-ARP1 maintained neutralizing activity *in vitro* when stored at room temperature for more than one year. After boiling at high temperature for 30 min, the rice water containing MucoRice-ARP1 (protein solution of MucoRice-ARP1 powder in PBS) lost only 33% of its binding capacity. In animal experiments, MucoRice-ARP1 was stable in the digestive tract of mice. It was shown to be detectable in the intestine of neonatal mice 6 h after ingestion. MucoRice-ARP1 exerted good prophylactic and therapeutic effects in mouse experiments compared to ingestion of PBS or wild-type rice by neonatal mice. Rotavirus-inoculated mice with prophylactic MucoRice-ARP1 presented a significantly lower rate of diarrhea and disease severity. They were also greatly alleviated when MucoRice-ARP1 was therapeutically ingested (Tokuhara et al., 2013).

Secretory IgA (SIgA) is the predominant protective antibody on the surface of the gastrointestinal mucosa. Vikram Virdi et al. replaced the antigen-binding fragment of IgA with a nanobody and produce in a seed-bioencapsulated SIgA-analog (sVHH-IgA) in model plant *Arabidopsis*. Seed extracts producing VHH-IgA antibodies inhibited bacterial binding to porcine gut villous enterocytes *in vitro*. Piglets on diets containing the VHH-IgA in the feed exhibited a progressive reduction in bacterial shedding and a significantly reduced immune response, confirming reduced exposure to enterotoxigenic *Escherichia coli* strains bearing F4 fimbriae (F4-ETEC). They also developed the monomeric VHH-IgA (mVHH-IgA) format. Piglets fed mVHH-IgA-containing diets such as Arabidopsis, soybean or Pichia exhibited good preventive and therapeutic effects against F4-ETEC (Virdi et al., 2013; Virdi et al., 2019). Similarly, nanobodies targeting the major outer membrane protein (MOMP) or flagellum of *Campylobacter* were fused to the constant domains of chicken IgA and IgY. The fused chimeric antibodies were efficiently expressed in leaves of Nicotiana benthamiana and in seeds of *Arabidopsis thaliana*. And these Nbs-containing *Arabidopsis* seeds are expected to contribute to in passive oral immunization of chickens (Vanmarsenille et al., 2018).

Nanobodies also present new ideas for passive immunity to prevent sexually transmitted diseases (STDs). It has been proposed that inoculation in the vagina with strains that express Nbs targeting specific pathogens holds promise for preventing infection with STDs, such as acquired immunodeficiency syndrome (AIDS). *Lactobacillus* rhamnosus DSM 14870 can colonize the human vagina and is clinically used as a vaginal capsule to treat bacterial vaginosis. Investigators constructed *Lactobacillus* rhamnosus DSM 14870 to express the HIV-neutralizing nanobody VHHA6 in soluble and surface covalently anchored forms. Both forms neutralized different subtypes of the primary HIV-1 strains *in vitro*, and binding occurred at an acidic pH in a vaginal-like environment. This model is expected to be inoculated against HIV in women at high risk for HIV. These novel passive immunization tools based on nanobodies contribute to reducing the economic burden of health care in the country and complement current vaccine-based prevention measures (Kalusche et al., 2020).

## Multivalent constructs of nanobody

**FIGURE 2:**
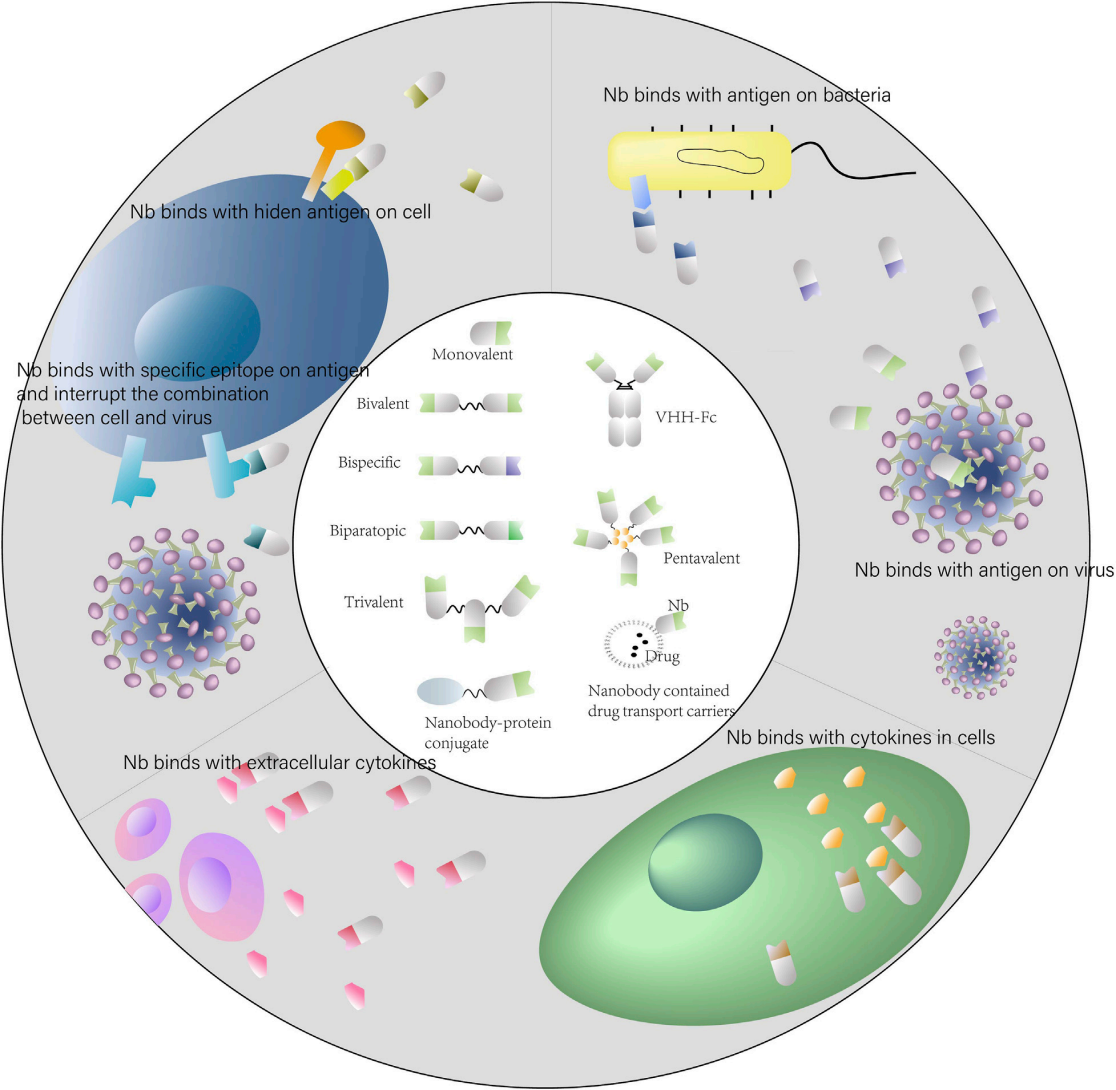
Applications of nanobodies in human infectious diseases. Schematic representation of the use of nanobodies and nanobody-derivatives for targeting infectious diseases.

Nanobodies possess advantages and excellent potential for applications in infectious diseases (Figure 2). However, their low molecular weight also results in a short half-life, which is not conducive for long-term treatment. This limitation could be partially circumvented by the construction of multivalent Nbs. The construction is relatively simple and versatile. Nanobodies have fully hydrophilic surfaces and do not contain light chains, allowing the construction of Bivalent or multivalent Nbs through linker peptides. A common linker peptide is the flexible glycine-serine linker (G4S), which tandemly associates different Nbs to form bivalent or multivalent Nbs. And the formed molecules often carry higher affinity or diverse specificity (Kostelny et al., 1992; Els Conrath et al., 2001; Bannas et al., 2017). In addition, it is also possible to construct multivalent Nbs using chemical bonds formed by interactions between amino acids. For example, nanobodies are fused to the hinge region through disulfide bonds formed by cysteine amino acid to construct dimers (Els Conrath et al., 2001). Polyvalent Nbs have a series of advantages. Multivalent constructs maintain the physicochemical properties, including high solubility, high yield and good stability. After lyophilization and aerosolization, they still exhibit potent neutralizing activity against pseudoviruses, displaying diverse drug delivery potential (Detalle et al., 2016). The affinity and viral blocking effect of multivalent Nbs would be significantly enhanced after constructed by homologous Nbs. Multi-specific Nbs are promising blocking the viral mutation evasion due to bind several epitopes inside the RBD of SARS-CoV-2 simultaneously (Xiang et al., 2020).

One example is nanobodies targeting SARS-CoV-2. SARS-CoV-2 binds to the human angiotensin-converting enzyme 2 (ACE2) receptor through the receptor-binding domain (RBD) to facilitate viral entry into cells. Thus, the RBD is a critical site for viral entry into cells and a key target for immunotherapy. Nanobodies bind with high affinity to the RBD and block further invasion. Huo et al. constructed two strains of nanobodies, H11-D4 and H11-H4, targeting the epitopes that partly overlapped with the ACE2 binding region. Constructed bispecific Nbs H11-H4-Fc extended the half-life and exhibited high potency, with a 50% neutralization dose (ND 50) of up to 4–6 nM (Huo J. D et al., 2021). Xiang et al. engineered several strains of Nbs targeting the SARS-COV-2 RBD region. In a pseudovirus luciferase assay, the half maximal inhibitory concentration (IC50) of the constructed homotrimeric Nbs was increased by approximately 30-fold compared to the monomeric version. Moreover, the heterodimeric constructs were up to four fold more potent than the monomeric form (Xiang et al., 2020). Similarly, another experiment indicated that, compared to monovalent Nbs, the binding capabilities of the homodimer were much better. Furthermore, the heterodimer exhibited increased RBD binding affinity and enhanced neutralizing ability against SARS-CoV-2 (Ma et al., 2021). Dong et al. constructed Nb heterogenous trimers showing stronger S1 binding affinity and effective SARS-CoV-2 pseudovirus neutralization than dimers or homotrimers (Dong et al., 2021).

Multivalent Nbs have been shown good applications in other infectious diseases. Bispecific Nbs targeting HIV gp41 and gp120 epitopes exhibit a potent neutralizing effect, with a 1400-fold enhancement in potency compared to a mixture of individual nanobodies (Strokappe et al., 2019). Nb ALX-0171 is a trimeric nanobody, which has already been in clinical trials. ALX-0171 targets Respiratory syncytial virus (RSV) with subnanomolar affinity and shows significant potential for the treatment of RSV-mediated disease (Detalle et al., 2016). A preclinical study demonstrated a significant >6000-fold increase in viral neutralization of RSV by ALX-0171 compared to the monovalent form. Moreover, a remarkable reduction in nasal and pulmonary viral load was observed in both prophylactic and therapeutic trials (Detalle et al., 2016). In phase I clinical trial, ALX-0171 was well tolerated by inhalation in over 100 adults, including airway-sensitive (hyperreactive) subjects, demonstrating good safety and pharmacokinetic properties (t_1/2_ ranged from 12.5 to 26.6 h) (Bruyn et al., 2015). A recent trial of ALX-0171 in hospitalized children with RSV lower respiratory tract infections has found that ALX-0171 has difficulty improving the clinical course of patients with RSV lower respiratory tract infections and is more conducive to early intervention before the onset of lower respiratory tract inflammation (Cunningham et al., 2021).

Nanobodies can also be constructed to target both pathogens and receptors *in vivo* in a multivalent form. Vlieger et al. constructed a bispecific nanobody targeting both the 23 amino acid residue-long M2 ectodomain (M2e) of influenza A virus and the Fc gamma receptor (FCγR) on human innate immune cells, which can be efficiently and economically expressed in Pichia pastoris cells and delivered intranasally to protect mice from lethal influenza A virus infection (De Vlieger et al., 2019).

*Shigella spp*., which cause bacillary dysentery, infect colonic epithelial cells through the Type III secretion systems (T3SS). Invasion plasmid antigen D (IpaD) is located on the T3SS and is a common therapeutic target for Shigella. Studies have shown that a Nb heterodimer formed by nanobodies targeting different epitopes of IpaD reduces the hemolytic activity of Shigella by >80%. In addition, VHHs usually recognize conformational epitopes and can be used to probe for the conformational changes and structure-function relationships of the targets. The investigators found that these IpaD epitope-specific Nbs was useful for the study of IpaD spatial structure as well as the structure-function relationship, which may be helpful for exploring novel therapeutics (Barta et al., 2017).

Rift Valley Fever virus (RVFV) and Schmallenberg virus (SBV) are two distinct infectious diseases. Paul J Wichgers Schreur et al. selected RVFV and SBV-specific Nbs that target the receptor-binding glycoprotein domains and created virus-neutralizing nanobody multimeric complexes using bacterial superglues, demonstrating that they were able to reduce virus-induced morbidity and mortality through prophylactic administration in mice. Furthermore, bispecific molecules that fuse nanobodies targeting RVFV and SBV with the Fc domain have been shown to perform well in therapeutic administration (Schreur et al., 2020).

Despite the superior performance of polyvalent Nbs and the maturity of antibody engineering techniques, different construction forms contribute to different functions of nanobodies. It remains challenging to design optimal nanobody combinations and forms (Iezzi et al., 2018; Schreur et al., 2020). De Tavernier’s research on bivalent Nbs shows that the modification of bivalent Nbs may have the following characteristics. Bispecific Nbs are superior to the bivalent form of a single nanobody. Combining the most therapeutically effective monovalent Nbs does not necessarily produce bispecific Nbs with superior performance, while a dual complementary site structure constructed from less therapeutically effective nanobodies may exert a better therapeutic effect (De Tavernier et al., 2016). Koenig and Das et al. attempted to design different multivalent Nb structures based on epitope mapping data from surface plasmon resonance (SPR), X-ray crystallography, and extensive conformational information of spike-Nb complexes determined by cryo-electron microscopy information (Koenig et al., 2021). Despite these promising results, building multivalent Nbs with excellent performance still requires a great deal of additional work.

## Nanobody conjugates and combination

Nanobodies are easy to be modified and engineered. Fusion proteins of nanobodies are often constructed in practical applications to enhance therapeutic effects. The VHH-Fc form, which is constructed by nanobody linked to IgG Fc region, is a common form. The VHH-Fc form can extend the half-life and enhance the antiviral effect, and also facilitate the Fc-mediated effector function (Burmistrova et al., 2016; De Vlieger et al., 2018; Laursen et al., 2018). Nick S et al. found that nanobody fusion proteins containing Fc showed a more potent effect against influenza virus (Laursen et al., 2018). The VHH-Fc form targeting *Mycoplasma* hominis also exerted effective preventive and therapeutic effects in mice model (Burmistrova et al., 2016). Nanobodies can also be combined with drug molecules to exert therapeutic synergy. For many drug molecules, specific transport to the therapeutic target is one of the significant challenges for drug therapy. For instance, small RNA-based molecules suffer from inefficient cellular uptake due to their negative charge and basically rely on their own hydrophilicity and small molecular weight to achieve drug delivery *in vivo*, which results in low transport efficiency, high drug consumption, and serious side effects (Cunha-Santos et al., 2020). Nanobodies are able to functioned as excellent candidates as tailored vehicles for molecules. As shown in Figure 2, the proteins fused with nanobodies can be specifically transported to its target for therapeutic efficacy and here are some concrete examples.

### Small interfering RNAs

Small interfering RNAs (siRNAs) that regulate posttranscriptional gene silencing have been validated for treating localized regions *in vivo*, such as intraocular and intracerebral diseases. Nonetheless, there are still limitations to the systemic delivery of therapeutic siRNAs. The therapeutic molecules formed by combining siRNA molecules with cell-penetrating peptides are too small to be rapidly cleared by the kidneys, leading to ineffective therapy. Nb-based constructs of VHH-siRNA chimeras target siRNA inhibitors to exert their inhibitory function and hold promise for treating a variety of diseases. C-X-C chemokine receptor type 4 (CXCR4) is widely expressed on CD4^+^ T lymphocytes, especially HIV-infected T lymphocytes. The interaction of CXCR4 with its natural ligand effectively mediates the onset of internalization. Cunha-Santos et al. constructed chimeras based on nanobodies targeting CXCR4 that aid siRNA translocation across membranes, rendering HIV viral transcription silent and inhibiting viral infectivity (Cunha-Santos et al., 2020).

### Immunotoxins

Immunotoxins are protein-based drugs. After bond with the specific cell-surface antigen, the Immunotoxins can internalize into the target cell and exert therapeutic effect (Khirehgesh et al., 2021). The conjugation of nanobodies with immunotoxins facilitates targeted delivery of the immunotoxin, contributing to more efficient killing of virally infected cells. Glycoprotein D (gD2), located on vaginal herpes simplex virus (HSV-2), is a key molecule in viral infection. The fusion of the Nb R33, which targets gD2 but is unable to neutralize the virus, with the cytotoxic domain of Pseudomonas aeruginosa exotoxin A produced an immunotoxin that was demonstrated to enhance toxin killing and killed virally infected cells more efficiently than the cytotoxic domain alone (Geoghegan et al., 2015). Apolipoprotein L-I (apoL-I) is able to lysis the African trypanosomes. Recombinant immunotoxin constructed by binding NbAn33, which targets trypanosomes, to apoL-I helped completely clear the parasite in mouse models without any therapeutic side effects (Baral et al., 2006).

### Drug molecules

For some drug molecules that have difficulty fusing directly with nanobodies to form fusion proteins to co-target therapeutic targets, nanobodies can be constructed as carriers for transporting these drugs. Targeted delivery enabled by nanobodies reduces the therapeutic dose of these drugs, enhances their efficacy, minimizes side effects, and avoids resistance mechanisms related to surface transporter protein mutations. African trypanosomiasis is a lethal disease in which constant mutation of the variant surface glycoprotein by antigenic variation prevents the host immune response and the implementation of vaccine immunization. At the same time, loss-of-function mutations in surface drug transport proteins accelerate the onset of drug resistance. Juan D. Unciti-Broceta et al. constructed a drug nanocarrier consisting of polymeric nanoparticles encapsulated with nanobodies targeting the surface of trypanosomes and carrying the trypanocidal drug pentamidine inside. The drug enters trypanosomes *via* endocytosis rather than through classical cell surface transport proteins. *In vitro* efficacy experiments demonstrated that treatment with pentamidine-loaded nanoparticles resulted in a lower IC50 than treatment with pentamidine alone, while *in vivo* treatment experiments in a mouse model showed that curing infected mice with the nanoparticles required a much lower drug dose than treatment with pentamidine alone (Unciti-Broceta et al., 2015).

### Vaccine antigens

Nanobodies can be used in combination with vaccines to enhance therapeutic efficacy. For example, post-exposure prophylaxis (PEP) against rabies infection consists of passive immunization with plasma-derived immunoglobulins and active immunization with a vaccine shortly after exposure. Because anti-rabies immunoglobulins are scarce and expensive, nanobodies are cheaper and effective alternative. The combination of anti-rabies nanobodies and a PEP vaccine for rabies delayed disease onset in intranasal rabies infection mice, extended the median survival time from 14 to 35 days, and increased the survival rate of mice from 19 to 60% compared to the treatment with nanobodies alone. In the combination treatment, similar to the side effects occurring with immunoglobulin, there was also some interference with the vaccine’s antigenicity by the anti-rabies nanobodies. However, it did not prevent the synergistic effect. The synergistic effect of anti-rabies Nbs and the vaccine in mice after rabies virus exposure further validates the potential use of anti-rabies Nbs for rabies PEP (Terryn et al., 2016). Vaccine antigens fused with Nbs specifically target antigen-presenting cells (APCs) to enhance their immune effect. For example, capsid protein-based virus-like particles (VLPs) trigger protective humoral immunity against papillomavirus (HPV) *in vivo* and prevent HPV infection and its subsequently induced malignancies. Targeted delivery of Ag to APCs by coupling E7 Ag of HPV16 to VHH targeting CD11b stimulates the generation of Ag-specific CD8^+^ T cells, elicits cellular immunity, induces cellular immune killing and treats tumors in HPV + positive patients. This VHH-Ag coupled vaccine elicits a greater antitumor CD8^+^ T cell response than the Ag vaccine alone and has been successfully used as a therapeutic vaccine in an HPV cancer model (Woodham et al., 2018). A SARS-CoV-2 vaccine constructed by fusing a nanobody targeting the class II major histocompatibility complex antigen with the SARS-CoV-2 spike RBD elicits potent humoral and cellular immunity in mice (Pishesha et al., 2021).

Amcheslavsky et al. proposed that the recognition of conserved epitopes through nanobodies were able to help reverse engineer useful vaccines aimed at Enterotoxigenic *Escherichia coli* (ETEC). They identified nanobodies that recognize a highly conserved epitope in the putative receptor-binding region of the ETEC adhesion proteins and proposed that rational design of ETEC vaccines based on this conserved epitope facilitated more effective disease prevention (Amcheslavsky et al., 2021).

## Nanobody targeting receptor proteins

Receptor proteins in the human body are also attractive targets for immunotherapy of infectious diseases. Antibodies can competitively bind receptor proteins and prevent the invasion of pathogens. However, some specific receptors with multiple functions in the body are involved in the process of viral infection invasion and are implicated in many critical physiological processes (Van Hout et al., 2018). Conventional antibodies are more likely to recognize chemical groups, peptides and epitopes located on large volumes of proteins with binding sites that exhibit cavities, grooves, or planar structures (Jovčevska and Muyldermans, 2020). It is hard for antibodies to aim at the small hidden epitopes on multifunctional receptors without interrupting other physiological responses. Therefore, directly targeted inhibition using traditional antibodies may cause severe side effects. Nanobodies can effectively bind to receptors and act. Thomas J et al. identified nanobodies that bind to human ACE2 and blocked the interaction of RBD and ACE2 with an affinity of 1–5 nM (Esparza et al., 2020). Besides, the longer CDR3 of the nanobodies form finger-like extensions that are able to bind with high affinity to small hidden epitopes in the concave surface of the antigen or in the antigen gap (Schmitz et al., 2013; Jovčevska and Muyldermans, 2020). Nanobodies are able to selectively bind to multifunctional epitopes on the receptor, blocking disease-related signaling pathways or responses while avoiding interference with the receptor’s normal function, thereby reducing the side effects of treatment.

CXCR4 is one of the G protein-coupled receptors (GPCRs) that is involved in many physiological responses, such as hematopoiesis, embryonic development of the cardiovascular and nervous systems, and leukocyte migration. However, CXCR4 is also a major coreceptor for HIV to enter CD4-positive T cell clusters and is an attractive target for HIV therapy. Although contradictory findings exist, former studies have also suggested that the HIV infection binding site on CXCR4 does not coincide with the normal physiological response site (Tian et al., 2005). Without precise targeting, conventional drugs targeting CXCR4 can interfere with the normal physiological functions of CXCR4 while treating HIV disease. In contrast, Nbs targeting CXCR4 treatment effect by, effectively inhibiting HIV while avoiding interference with the normal physiological functions of CXCR4. Van Hout and his colleagues identified a nanobody that recognized an epitope on CXCR4 that is distinct from other ligand-binding epitopes and interacting with reported chemokine receptors (Van Hout et al., 2018). Such a study also demonstrates the great potential of nanobodies for reducing the side effects of immunotherapy.

## Nanobody targeting pathogens

There are many different steps during the life cycle of pathogens in the body, including cell attachment and entry, replication and regulation, transcription and translation, and virion assembly, offering diverse targets (Koromyslova et al., 2020; Tsukuda and Watashi, 2020). Pathogens have low homology with humans, exhibiting a variety of attractive epitopes. Immunotherapy has shown great potential for treating infectious diseases, but several obstacles remain. One of these difficulties is that many key epitopes on pathogens are well modified to form complex spatial conformations or hidden under other molecules, such as glycoproteins, avoiding recognition by conventional antibodies. These epitopes include those that bind to receptors on human cells, which mediate pathogen entry into the body or cells and essential factors of virulence for pathogens to exert virulence, etc. (Lutje Hulsik et al., 2013; King et al., 2018). Ultimately, it prevents the production of effective therapeutic antibodies after pathogen infection. On the other hand, the constant appearance of new variations presents a significant challenge to infection therapy, especially viral infection therapy. In that case, most antibodies aimed at epitopes on pathogens cannot provide adequate protection for different subtypes or against future strains. Fortunately, antibodies with broad neutralization and combination activity across different subtypes of viruses have been discovered. These antibodies bind highly conserved epitopes on the virus. Studies have revealed that such antibodies are rare because viruses try to hide their conserved determinants of pathogenicity, making them difficult to recognize by conventional antibodies. Nanobodies exhibit good potential for applications in this area. Compared to conventional antibodies, they have a lower molecular weight and a longer CDR3 region, which is beneficial to higher affinity and identification ability. Therefore, Nbs are more suitable for binding to these conserved epitopes hidden in pockets or clefts. Nbs are expected to be the preferred mode to attack pathogens across divergent subtypes or even future subtypes (Wei et al., 2011; Liu et al., 2015; Gao et al., 2020).

### Norovirus

Nanobodies inhibit pathogen infection through multiple mechanisms of action, such as inhibiting viral entry into cells or inhibiting toxins secreted by pathogens to keep them from harming the body. Human norovirus is one of the major causes of gastroenteritis. There are no vaccines or antivirals for norovirus thus far. Structural studies for norovirus have indicated that the protruding (P) domains contain host binding factors’ main determinants. Histo-blood group antigens (HBGAs) are important binding factors in the P domain. Researchers screened a large nanobody library for norovirus and obtained a panel of nanobodies binding with different epitopes. Some Nbs directly overlap with the HBGA binding sites. These nanobodies can compete with the receptor, which eventually blocks virion binding sites. Moreover, some Nbs cause structural damage to the virions to inhibit their binding. Nanobodies that bind at a dimeric interface on the lower side of the P dimer disrupt a structural change in the capsid associated with binding cofactors. Furthermore, nanobodies induce conformational rearrangement of several P domain loops, leading to particle aggregation and interference at the HBGA binding pocket (Koromyslova and Hausman, 2018; Koromyslova et al., 2020). Kerstin Ruoff et al. also demonstrated that nanobodies have different targets and inhibit norovirus infection through various mechanisms. Furthermore, the therapeutic effects are improved by combining treatment with different nanobodies recognizing multiple epitopes (Ruoff et al., 2019).

### Plasmodium falciparum

Plasmodium falciparum causes placental malaria, resulting in adverse outcomes for mothers and children. The plasmodium-falciparum-expressed protein Variant surface antigen 2-CSA (VAR2CSA) binds to chondroitin sulfate A (CSA) in the human placenta, promoting infected erythrocytes (IE) accumulation. Antibodies targeting CSA-binding epitopes on VAR2CSA competitively block the binding of VAR2CSA to CSA and effectively prevent the adhesion of IE. However, VAR2CSA is large and structurally complex, and the epitope region responsible for binding to CSA is small and hidden. Therefore, it is challenging to identify antibodies that specifically target the CSA-binding region on VAR2CSA. Nanobodies recognize cryptic, conformational epitopes that are inaccessible to conventional antibodies and interact with smaller immunogenic epitopes, representing effective alternative to conventional antibodies that bind nonimmune dominant epitopes. Sisse B. Ditlev et al. directly immunized alpacas with full-length VAR2CSA (FV2), but not immunodominant domains of VAR2CSA, to screen for Nbs targeting VAR2CSA. They acquired nanobodies that specifically recognized different nonimmune dominant and hidden epitopes on VAR2CSA, including the CSA-binding epitope region. These antibodies are expected to play an essential role in disease research, diagnosis and treatment in the future (Ditlev et al., 2014).

### *Listeria* monocytogenes

*Listeria* monocytogenes is a food-borne pathogen that poses a high risk to pregnant women and may lead to termination of fetal development. The interaction of internalin B (InlB) with the c-Met receptor is essential for bacteria to cross the placental barrier in pregnant women. InlB is hidden in the peptidoglycan layer, which is difficult for conventional antibodies to bind and inhibit. A previous study showed that Nbs are small enough to penetrate the *listeria* cell to bind the target protein. They can bind to the c-Met receptor-binding site on InlB with high affinity and competitively inhibit bacterial invasion, preventing fetal infection (King et al., 2018).

### Influenza

Seasonal influenza viruses continue to cause epidemics around the world each year, resulting in severe death and social panic. Moreover, due to the rapid accumulation of genetic mutations, influenzas evolve frequently and have a variety of subtypes. Ongoing mutations also make novel influenza pandemics frequent and unpredictable. Unpredictable outbreaks of influenza cause serious concern about possible pandemics in the future. Effective prophylactic and therapeutic methods are urgently needed.

Broadly neutralizing antibodies against influenza provide an attractive idea for the broad-spectrum treatment of influenza viruses. The M2 ion channel contains an extracellular domain that is conserved across all human influenza A viruses and is a potential target site for broad-spectrum treatment. However, due to the smaller number and low immunogenicity of M2 molecules revealed on influenza viral particles, there is currently no influenza vaccine that targets this epitope. Nanobody M2-7A targets the M2 ion channel on the virus with a good affinity. It shows potent neutralizing activity against multiple subtypes of influenza A viruses, demonstrating broad-spectrum neutralization potential (Wei et al., 2011).

Hemagglutinin (HA) is one of the key targets for drugs and vaccines against influenza viruses. It plays a critical role in mediating virus-host cell receptor binding and virus-host cell membrane fusion (Gao et al., 2020). Hufton et al. identified several nanobodies targeting the HA protein domain of influenza viruses that exhibited strong cross-neutralization potential.

One of these nanobodies, named R1A-B6, presents five cross-neutralizing potential and binds to H1N1 and H5N1 with IC50 values in the single-digit nanomolar range. Studies have indicated that constructing these nanobodies into bivalent or multivalent forms could further improve neutralizing activity and therapeutic efficacy (Hufton et al., 2014; Gaiotto and Hufton, 2016).

### HIV

The GP41 glycoprotein on HIV-1 contains a highly conserved segment within the membrane-proximal external region (MPER) and is a common target for HIV neutralizing antibodies. However, due to the unique spatial conformation of MPER and the multiple evasion strategies of the virus, conventional immunization protocols using gp41 peptides or protein immunization have difficulty producing broad-spectrum cross-neutralizing antibodies targeting MPER. Some investigators immunized alpacas with gp41-MPER proteoliposomes and identified the MPER-specific nanobody 2H10.2H10 is capable of binding to epitopes with low nanomolar affinity in various particle conformations. Crystal structure studies revealed that 2H10 has a longer CDR3 region with solvent-exposed tryptophan at its tip. The constructed bivalent Nb bi-2H10 achieved better immunosuppression by simultaneously binding two GP41 trimers or by interacting with two GP41 molecules on the same trimer (Lutje Hulsik et al., 2013).

### HPV

Many pathogens invade cells and proliferate inside them, causing a series of intracellular reactions that damage the body and evade external immune mechanisms. Conventional antibody drugs are difficult to successfully deliver to cells and maintain functions. Due to the small molecular size, nanobodies can be expressed as “intracellular antibodies”, also called as intrabodies, that target intracellular antigens to effectively and specifically inhibit their functions and exert immune effects.

Nb-based intrabodies exhibit initially successes in the treatment of human papillomavirus (HPV). Persistent infection with HPV may result in the development of cervical cancer. HPV16 is a common type of HPV infection in humans. Moreover, E6 and E7 proteins of HPV16 play key roles in tumor aggressiveness. Researchers identified nanobodies that bind with E6 and E7, named Nb9 and Nb2. Both inhibited tumor cell proliferation *in vitro* when expressed in HPV16-positive cells as intracellular Nbs. Researchers subsequently constructed animal models to study the therapeutic effects of using Nb9 alone or combining Nb9 and Nb2. The *in vivo* experiments indicated that both therapeutic modalities inhibited tumor growth, but the combination therapy was more effective. These experimental results demonstrate the potential value of nanobodies as intrabodies for the treatment of HPV-associated lesions (Li et al., 2019; Zhang et al., 2021b).

### Other pathogens

It is hard to treat infection with intracellular obligate pathogens due to the intracellular targets and delivery systems. *Ehrlichia* translocated factor-1 (Etf-1) is the major virulence factor of the intracellular bacterial *Ehrlichia* chaffeensis. Secreted Etf-1 anchors mitochondria and prevents host cells apoptosis mediated by mitochondria. Infected host cells will continue to survive, thus providing a site for intracellular replication of the bacteria. Nanobodies were found to enter the cell and specifically interfere with the mitochondrial localization of Etf-1, thereby blocking Etf-1 function and thus preventing Chaffeensis infection in cells and mouse models (Zhang et al., 2021a). The porcine reproductive and respiratory syndrome virus (PRRSV) is a highly antigenically variable virus for which effective antibodies are difficult to obtain. Nanobodies targeting specific epitopes of PRRSV strongly inhibited or even entirely prevented PRRSV replication by inhibiting viral genome replication and transcription, preventing virus-induced cytopathic changes in cells (Liu et al., 2015).

Nanobodies targeting conserved epitopes on the major outer membrane protein of *Campylobacter* jejuni and *E. coli*. also show potential therapeutic and diagnostic effects (Vanmarsenille et al., 2017).

In these applications, nanobodies have demonstrated their superiority over traditional antibodies with respect to their ability to bind key epitopes typically hidden by pathogens for therapeutic action.

## Nbs binding regulators

Nanobodies also bind immune factors in the body and participate in the immune regulation of diseases. Septic shock is one of the leading causes of death in intensive care patients. Macrophage migration inhibitory factor (MIF), released by myeloid cells, is an upstream immunomodulatory mediator involved in the mechanisms leading to infections and septic shock and is an attractive therapeutic target. Nanobodies can significantly bind to MIF with nanomolar affinities, inhibit MIF activity and reduce endotoxic shock–mediated lethality in mouse models (Sparkes et al., 2018).

The regulator of expression of virion protein (REV) is an essential transcriptional activator that regulates HIV gene replication. It also mediates the transport of HIV mRNAs from the nucleus to the cytoplasm by multimerizing on the secondary stem–loop structure of viral intron-containing mRNAs. A nanobody named Nb190 strongly inhibits REV multimerization, ultimately impeding the production and spread of new HIV viruses. Boons et al. directly transfected plasmids containing the Nb190 sequence into cells and constructed cell lines that stably expressed Nb190. Experiments revealed that these cells relatively avoided virus-induced cytopathological changes. Sequence analysis showed that the sites targeted by Nb190 in REV were well conserved among the major HIV-1 subtypes. Thus, Nb190 has the potential for broad-spectrum inhibition against different HIV-1 subtypes (Boons et al., 2014).

## Conclusion

Infectious disease is a severe threat to human health worldwide. The rampant spread of COVID-19 and other infectious diseases has necessitated increased requirements for the prevention and treatment of infectious diseases. Our review of the recent study shows that nanobodies, multi-specific drugs, and imaging reagents built with nanobodies represent an alternative anti-infection therapeutic opportunity against bacterial and viral outbreaks. Nanobodies has excellent tissue penetration, high affinity, flexible structure, and a cost-effective expression system, which provides new ideas and breakthroughs for preventing and treating infectious diseases. Nanobodies are expected to have broad application potential in various aspects of infectious diseases, such as inhibition of viral transcriptional replication and cell entry, neutralization of viral toxins, stimulation of the human immune system, and killing of virus-infected cells. Nanobodies have attracted increasing attention for applications in the prevention and treatment of bacterial toxins, traditional malignant infectious diseases and acute infectious diseases. Recent accumulated clinical and experimental data suggest that multimeric and functionalized molecules built with nanobodies will become a significant component of future diagnostic and therapeutic reagents, especially in infectious diseases. However, as a new type of antibody, there is still much to learn before translating the basic research results of nanobodies into applied products and gradually moving toward the clinic, requiring the joint efforts of future researchers.

In summary, the applications of nanobodies for the prevention, detection, and treatment of a wide variety of infectious diseases will improve human life and health, respond to public health events of infectious diseases, and facilitate a more economical and efficient public health system.
